# Reduction–Oxidation Photocycle Dynamics of Flavins in Starch Films

**DOI:** 10.3390/ijms13079157

**Published:** 2012-07-23

**Authors:** Alfons Penzkofer

**Affiliations:** Faculty of Physics, University of Regensburg, Universitaetsstrasse 31, D-93053 Regensburg, Germany; E-Mail: alfons.penzkofer@physik.uni-regensburg.de; Tel.: +49-941-943-2107; Fax: +49-941-943-2754

**Keywords:** riboflavin, lumiflavin, starch, oxystarch, flavin photo-reduction cycle, starch photo-oxidation cycle, static fluorescence quenching, photo-induced starch restructuring, phosphorescence, delayed fluorescence

## Abstract

The blue-light photo-reduction (conversion of oxidized flavin quinone via flavin semiquinone to fully reduced flavin hydroquinone) and dark re-oxidation of the flavins riboflavin and lumiflavin in starch (α-amylose) films was studied by absorption and luminescence spectroscopy. Blue-light photo-excitation caused an absorption, fluorescence, and phosphorescence decrease which recovered in the dark. The photo-reduction dark-oxidation cycle could be repeated. The efficiency of photo-reduction decreased with exposed excitation energy, and the speed of re-oxidation in the dark slowed down with time after excitation. The absorption did not fully recover. The fluorescence efficiency after a long time of storage in the dark increased beyond the initial flavin quinone fluorescence efficiency. Flavin photo-excitation is thought to cause starch-flavin restructuring (static fluorescence quenching center formation), enabling enhanced photo-induced starch to flavin electron transfer with subsequent flavin reduction and starch oxidation. In the dark, after light switch-off, thermal reversion of flavin reduction and starch oxidation occurred.

## 1. Introduction

**Chart 1:**
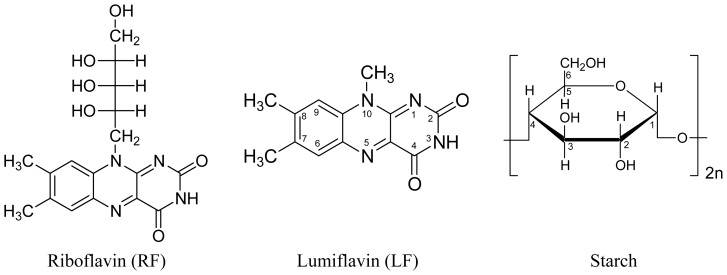
Structural formulae of riboflavin (RF), lumiflavin (LF), and anhydrous glucose repeat unit of starch (α-amylose).

Riboflavin (vitamin B_2_, lactoflavin, 7,8-dimethyl-10-ribityl-isoalloxazine, structural formula in Chart 1) and lumiflavin (7,8,10-trimethyl-isoalloxazine, structural formula in Chart 1) belong to the huge family of flavins [1–4]. Riboflavin plays an important role as cofactor in enzymes [1]. Lumiflavin is the core molecule of the flavins (isoalloxazines). It is the dominant photoproduct of riboflavin, flavin mononucleotide (FMN, riboflavin-5′-monophosphate) and flavin adenine dinucleotide (FAD, riboflavin-5′-adenosine-diphosphate) in alkaline solution (pH > 9) [2].

The photochemistry of riboflavin in different solvents is very rich with different irreversible and reversible degradation products ([2–7] and references therein). Intra-molecular hydrogen abstraction from the ribityl-side chain was found to play a dominant role in the isoalloxazine core reduction [5]. The photochemistry of lumiflavin and lumiflavin derivatives was studied in [8–10]. The one-electron and two-electron reduction modes of the flavin chromophore were clarified by flash photolysis studies on 3-methyllumiflavin and lumiflavin-3-acetic acid in aqueous solution [11].

FMN and FAD are the chromophores (cofactors) in blue-light sensitive photoreceptors [12–14]: FMN in LOV domains of phototropin [15], FAD and FMN in BLUF proteins [16], FAD in cryptochromes and photolyases [17]. The flavins exist in three redox states which are the flavin quinones (fully oxidized flavin) Fl, the flavin semiquinones (semi-reduced flavin) FlH^·^, and the flavin hydroquinones (fully reduced flavin) FlH_2_ [1–4]. The nature of the host determines the flavin redox state. Depending on the pH of aqueous solutions each of theses flavin redox species may exist in cationic, neutral, or anionic form [1–4]. In LOV domains of phototropins, FMN in oxidized neutral form is the cofactor, and the photocycle dynamics is determined by the reversible photo-induced formation of FMN-C4a-cysteinyl adduct [15,18]. In BLUF proteins, photo-induced electron transfer from tyrosine to neutral oxidized FAD or FMN leads to radical anion (FAD^·−^ or FMN^·−^) formation (flavin quinone radical anion) which causes reversible hydrogen-bond restructuring and possible reduction (proton transfer) to FADH^·^ or FMNH^·^ (flavin semiquinone) and further reduction by electron transfer to FADH^−^ or FMNH^−^ (anionic flavin hydroquinone) [19,20]. In cryptochromes, photocyclic reduction of neutral oxidized FAD was observed (first step is FAD^·−^ radical anion formation by photo-induced electron transfer) [21–26]. In photolyases, fully reduced anionic FADH^−^ is photo-oxidized to neutral flavin semiquinone FADH^·^ by electron release in the photo-induced DNA repair mechanism [17].

In a recent study on the luminescence (fluorescence, phosphorescence and delayed fluorescence) of riboflavin and lumiflavin starch films as hosts had been used (flavins in solid matrix, structural formula of anhydrous glucose starch repeat unit is shown in Chart 1) in order to be able to carry out the measurements with a conventional fluorimeter at room temperature [27]. A fluorescence decrease had been observed during the measurements indicating low fluorescence stability of the samples to light exposure. This observation initiated the light dependence investigations on riboflavin and lumiflavin presented in this paper.

We observed photocyclic behavior of riboflavin (RF) and lumiflavin (LF) in starch host. Neutral fully oxidized RF and LF were reversibly photo-reduced to RFH_2_ and LFH_2_, respectively. It is thought that thereby starch is reversibly oxidized to oxystarch [28–31]. The absorption and luminescence showed photocyclic behavior since flavin quinone, flavin semiquinone and flavin hydroquinone have different absorption spectra, and flavin semiquinone as well as flavin hydroquinone are non-luminescent. Static quenching [32–35] of the flavin quinone fluorescence occurred (reduction of quantum yield without change of lifetime) by photo-induced electron transfer from starch to appropriate arranged flavin molecules. The photo-excitation caused starch-flavin restructuring towards enhanced static fluorescence quenching. After photo-excitation, in the dark at room temperature re-oxidation of flavin hydroquinone to flavin quinone occurred and static fluorescence quenching decreased. The photo-induced flavin reduction and dark re-oxidation was repeatable (photocycle behavior). The photocyclic behavior of flavin implies a starch oxidation (oxystarch formation [28–31]) during light exposure and back reduction in the dark.

## 2. Results and Discussion

### 2.1. Absorption Photocycling

**Figure 1:**
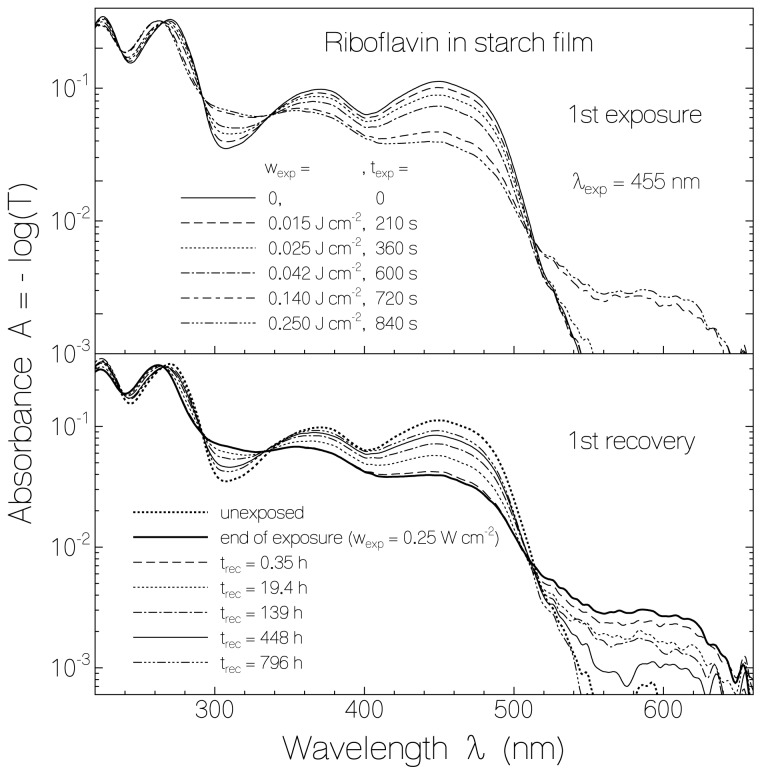
Absorbance spectra of riboflavin in starch film. The development of absorbance spectra during light exposure at wavelength λ_exp_ = 455 nm is shown in the top part. The bottom part shows absorbance spectra recovery after excitation light switch-off.

For RF in a starch film the absorption changes due to light exposure at 455 nm (top part, previously unexposed sample) and the absorption behavior after light switch-off in the dark (bottom part) are shown in Figure 1. The excitation intensity was varied with filters, and after certain times of exposure the transmission spectra were measured. The accumulated exposed energy densities, *w*_exp_, and the corresponding accumulated times of exposure, *t*_exp_, are listed in the top part of Figure 1. The RF absorption around 450 nm (first absorption band of fully oxidized RF) and around 371 nm (second absorption band of fully oxidized RF) decreased with exposed excitation energy density (with time of light exposure). A weak absorption band in the range from 515 nm to 660 nm built up which is attributed to the formation of semi-reduced riboflavin RFH^·^. The increasing absorption around 310 nm and 260 nm with isobestic points at 337.5 nm, 292 nm, 265 nm, and 237 nm is attributed to the formation of fully reduced riboflavin RFH_2_. Absorption cross-section spectra of flavins in fully oxidized form [36–38], semi-reduced form [39–41], and fully reduced form [42,43] are found in the cited literature. Re-oxidation of RFH· and RFH_2_ to RF in the dark is clearly seen in the lower part of Figure 1. The re-oxidation is not complete (some permanent reduction occurred). The absorption spectrum of the re-oxidized RF is not fully identical with the initial unexposed RF. The first three absorption maxima changed from 450 nm, 371 nm, and 271 nm to 449 nm, 369 nm, and 268 nm, respectively. The differences indicate local interaction changes between riboflavin and starch in the initial unexposed form and the recovered form.

**Figure 2:**
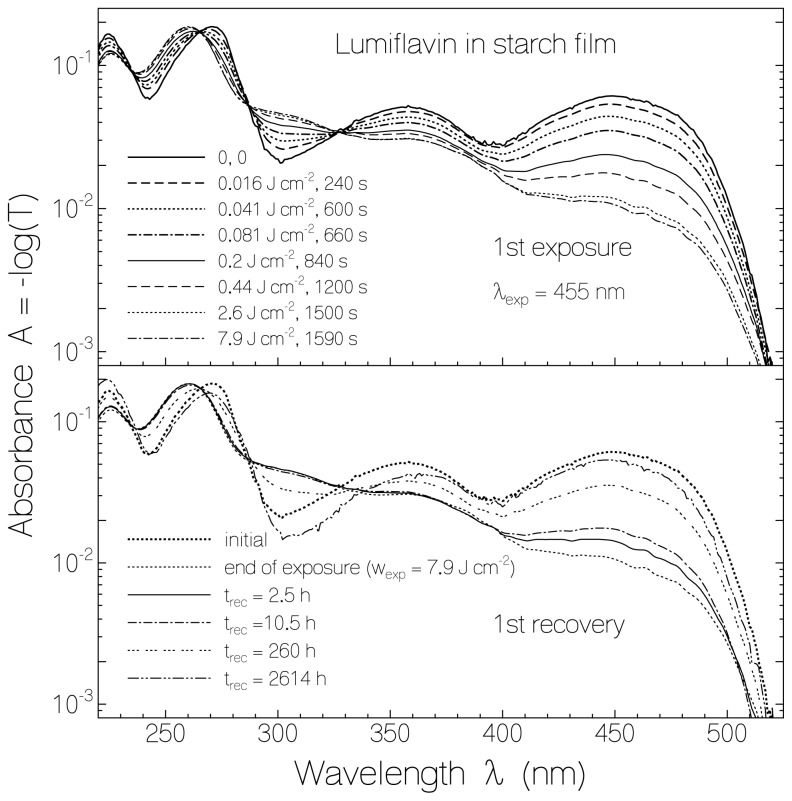
Absorbance spectra of lumiflavin in starch film. The development of the absorbance spectra during light exposure at λ_exp_ = 455 nm is shown in the top part. The bottom part shows the absorbance spectra recovery after excitation light switch-off.

For LF in a starch film, the absorption changes due to light exposure at 455 nm (previously unexposed sample) and the absorption behavior after light switch-off in the dark are shown in Figure 2. The photo-reduction of LF to LFH_2_ is seen in the top part. The accumulated exposed energy densities, *w*_exp_, and the corresponding accumulated times of exposure, *t*_exp_, are listed in the figure. The formation of semi-reduced lumiflavin could not be resolved (likely due to faster reduction of LFH· to LFH_2_). The re-oxidation of LFH_2_ to LF in the dark is seen in the lower part of Figure 2. The initial absorption spectrum of LF is not fully restored in the re-oxidation process. The positions of the first three absorption maxima of LF changed from 449 nm, 358 nm, and 271 nm to 448 nm, 366 nm and 270 nm due to local interaction changes between lumiflavin and starch in the photo-excitation process.

**Figure 3:**
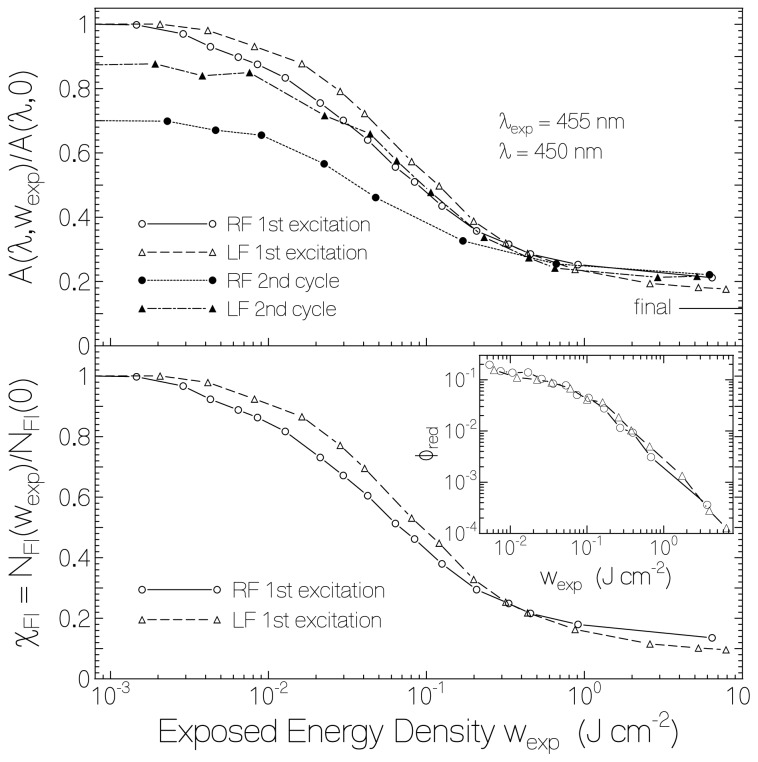
Top part: Dependence of normalized absorbance at λ = 450 nm *versus* exposed excitation energy density, *w*_exp_, at λ_exp_ = 455 nm. Bottom part: Mole-fraction of retained flavin in fully oxidized state, *χ*_Fl_, *versus* exposed excitation energy density. Inset in bottom part: Exposed energy density dependent quantum yield of photo-reduction, *φ_red_*, of Fl to FlH_2_.

In the top part of Figure 3 the dependence of absorbance at λ = 450 nm as a function of exposed energy density at λ_exp_ = 455 nm is shown for RF in starch (solid line connected circles, fresh previously unexposed sample) and LF in starch (dashed line connected triangles, fresh previously unexposed sample). Absorbance ratios A(λ = 450 nm, *w*_exp_)/A(λ = 450 nm, *w*_exp_ = 0) are displayed. The data for RF in starch belong to another film than used in Figure 1 (film exposed in more steps up to higher total exposed energy density, initial absorbance *A*(λ = 450 nm, 0) = 0.0692). The data for LF in starch belong to the curves presented in Figure 2. The horizontal line at the right hand side indicates the expected final absorbance ratio for the case of complete reduction of RF and LF to RFH_2_ and LFH_2_, respectively. The efficiency of photo-reduction was largest at the beginning of sample exposure and leveled off with continued exposure (see logarithmic abscissa).

The dotted-line connected dots in the top part of Figure 3 belong to a second photo-excitation of RF in starch. The sample was re-exposed after recovering in the dark for a period of 4 month. At the start of second exposure the absorbance had been recovered to about 70% of its original unexposed value. The absorbance ratio at the end of second exposure (*w*_exp_ = 7 J·cm^−2^) was approximately the same as at the end of the first exposure. The dash-dotted line connected filled triangles belong to a second photo-excitation of LF in starch. The sample was re-exposed after recovering in the dark for a period of 5 month and 20 days. At the start of second exposure the absorbance had been recovered to about 87% of its original unexposed value. The absorbance ratio at the end of second exposure (*w*_exp_ = 5.2 J·cm^−2^) was slightly higher than at the end of the first exposure. In the second excitation process of LF in starch some lumiflavin semiquinone formation was observed (long-wavelength absorption).

The lower part of Figure 3 displays the mole-fraction of still oxidized flavin during the first excitation cycle, $\chi_{Fl}(w_{\text{exp}})={\text{N}}_{Fl}(w_{\text{exp}})/{\text{N}}_{Fl}(0)=\int_{0}^{\ell }N_{Fl}(w_{\text{exp}},x)dx/\int_{0}^{\ell }N_{Fl}(0,x)dx$, where N_Fl_ is the length-integrated number density of oxidized flavin, *N*_Fl_(*x*) is the number density of oxidized flavin at coordinate *x*, and ℓ is the film thickness. *χ*_Fl_ is related to the absorbance ratio, *A*(*w*_exp_ )/*A*(0), by

(1) $$\chi_{Fl}(w_{\text{exp}})=\frac{\frac{A(w_{\text{exp}},\lambda )}{A(0,\lambda )}-\frac{\sigma_{a,{FlH}_{2}}(\lambda )}{\sigma_{a,Fl}(\lambda )}}{1-\frac{\sigma_{a,{FlH}_{2}}(\lambda )}{\sigma_{a,Fl}(\lambda )}}$$

Equation (1) is obtained from the relations, *A*(*w*_exp_) = {*χ_Fl_* (*w*_exp_)*σ_a_*_,_ *_Fl_* + [1 − *χ_Fl_* (*w*_exp_)]*σ_a_*_,_ *_FlH_*__2__}N*_Fl_*(0)/ln(10), and *A*(0) = σ*_a_*_,_*_Fl_* N *_Fl_* (0)/ln(10). *χ_FlH_*__2__ = 1 − *χ_Fl_* is the mole-fraction of fully reduced flavin. λ is the probe wavelength. In the calculations σ_a,Fl_(λ = 450 nm) ≈ 4.7 × 10^−17^ cm^2^ [36], and *σ_a_*_,_*_FlH_*__2__ (λ = 450 nm) ≈ 4.18 × 10^−18^ cm^2^ [43] was used. The retained mole-fraction of flavin quinone at the end of exposure (*w*_exp_ ≈ 8 J cm^−2^) was *χ*_RF,end_ ≈ 0.135 and *χ*_LF,end_ ≈ 0.096.

The momentary exposed energy density dependent quantum efficiency of photo-reduction, *φ_red_*, may be determined by use of the relation [43],

(2a) $$\varphi_{red}=\frac{\Delta {\text{N}}_{Fl}}{\Delta n_{ph,abs}}$$

where ΔN_Fl_ is the length-integrated number density of oxidized flavin which got reduced due to the number density of absorbed photons Δ*n*_ph,abs_.

ΔN_Fl_ is given by

(2b) $$\Delta {\text{N}}_{Fl}=\Delta \chi_{Fl}{\text{N}}_{Fl}(0)=[\chi_{Fl}(w_{\text{exp},1})-\chi_{Fl}(w_{\text{exp},2})]{\text{N}}_{Fl}(0)$$

where N*_Fl_* (0) is the initial length-integrated number density of oxidized flavin. It may be expressed in terms of initial absorbance, *A*(0,λ), and flavin quinone absorption cross-section, σ_a,Fl_(λ), at wavelength λ as

(2c) $${\text{N}}_{Fl}(0)=\frac{A(0,\lambda )\text{ln}(10)}{\sigma_{a,Fl}(\lambda )}$$

(Note that $A=-\text{log}(T)=\sigma_{a}\int_{0}^{\ell }N(x)dx/\text{ln}(10)$).

Δ*n*_ph,abs_ is given by

(2d) $$\Delta n_{ph,abs}=\frac{\Delta w_{\text{exp}}}{h\nu_{\text{exp}}}(1-{\bar{T}}_{\text{exp}})\approx \frac{w_{\text{exp},2}-w_{\text{exp},1}}{h\nu_{\text{exp}}}\left(1-\frac{10^{-A_{1}(\lambda_{\text{exp}})}+10^{-A_{2}(\lambda_{\text{exp}})}}{2}\right)$$

*A*_1_(λ_exp_) and *A*_2_(λ_exp_) are the absorbance values belonging to the exposed energy densities *w*_exp,1_ and *w*_exp,2_, respectively. *hν_exp_* is the excitation photon energy (*h* Planck constant, *ν_exp_* = *c*_0_/*λ_exp_* excitation frequency, *c*_0_ vacuum light velocity). &*Tmacr;*_exp_ ≈ [*T* (*w*_exp,1_ + *T* (*w*_exp,2_)] / 2 is the mean transmission at the excitation wavelength λ_exp_ in the excitation energy density interval of *w*_exp,1_ to *w*_exp,2_. Using adjacent data points of *w*_exp_, *χ*_Fl_, and *A*, the *φ_red_* (*w_exp_*) curves were determined for RF and LF which are displayed in the inset of the lower part of Figure 3. The initial quantum yields of photo-reduction were *φ_red_* (RF/starch, *w*_exp_→0) = 0.2 ± 0.03 and *φ_red_* (LF/starch, *w*_exp_→0) = 0.12 ± 0.03. At the end of exposure the quantum yield of photo-reduction lowered to *φ_red_* ≈ 10^−4^ for both samples (less and less favorable sites for flavin reduction and starch oxidation, part of Δ*n*_ph,abs_ belongs to FlH_2_ absorption).

**Figure 4:**
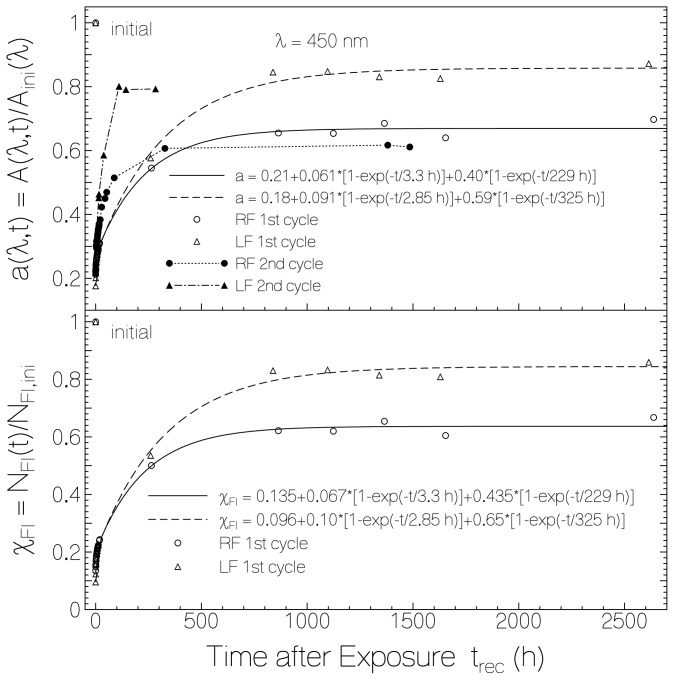
Top part: Dependence of normalized absorbance, *a* = *A*/*A*_ini_, at λ = 450 nm *versus* time *t*_rec_ after end of exposure. Bottom part: Mole-fraction of flavin in fully oxidized state, *χ*_Fl_, *versus* time after end of first exposure.

The absorbance recovery of flavin in starch after light exposure is manifested in Figure 4. In the top part of Figure 4, the circles belonging to RF in starch and the triangles belonging to LF in starch show the absorbance recovery in the dark after the first light exposure. The corresponding points in the bottom part of Figure 4 show the mole-fractions of oxidized RF and oxidized LF. The absorbance (mole-fraction) of RF in starch recovered back to ≈70% (67%) of the initial unexposed value. In the case of LF in starch the back recovery reached ≈86% (85%) of the initial unexposed value.

The absorption recovery and the flavin re-oxidation fit reasonably well to bi-exponential functions according to

(3a) $$a(\lambda ,t_{rec})=\frac{A(\lambda ,t_{rec})}{A_{ini}(\lambda )}=a(\lambda ,0)+[1-a(\lambda ,0)]\{\kappa_{a,f}[1-\text{exp}(-t_{rec}/\tau_{rec,f})]+\kappa_{a,s}[1-\text{exp}(-t_{rec}/\tau_{rec,s})]\}$$

(3b) $$\chi_{Fl}(t_{rec})=\frac{{\text{N}}_{Fl}(t_{rec})}{{\text{N}}_{ini}}=\chi_{Fl}(0)+[1-\chi_{Fl}(0)]\{\kappa_{\chi ,f}[1-\text{exp}(-t_{rec}/\tau_{rec,f})]+\kappa_{\chi ,s}[1-\text{exp}(-t_{rec}/\tau_{rec,s})]\}$$

as is seen by the solid and dashed curves in Figure 4. *a*(λ,0) = *A*(λ, *t* = 0) / *A_ini_* (λ) is the absorbance ratio at wavelength λ and time *t*_rec_ = 0 after end of exposure. *χ*_Fl_(0) is the mole-fraction of retained oxidized flavin at end of exposure. τ_rec,f_ is the time constant of fast absorption recovery, and τ_rec,s_ is the time constant of slow absorption recovery. The coefficients of fast recovery, κ_a,f_ and κ_χ,f_, and the coefficients of slow recovery, κ_a,s_ and κ_χ,s_, are related by Equation (1). The fraction of photo-reduced flavins which retained permanently reduced is: *κ* *_χ_*_,_ *_per_* = 1 − *κ_χ_*_,_ *_f_* − *κ_χ_*_,_ *_s_*. The obtained parameters for RF in starch are χ_RF_(0) = 0.135, κ_χ,f_(RF) = 0.077, τ_rec,f_(RF) = 3.3 h, κ_χ,s_(RF) = 0.50, τ_rec,s_(RF) = 229 h, and κ_χ,per_(RF) = 0.423. The obtained parameters for LF in starch are χ_LF_(0) = 0.096, κ_χ,f_(LF)= 0.11, τ_rec,f_(LF) = 2.85 h, κ_χ,s_(LF) = 0.72, τ_rec,s_(LF) = 325 h, and κ_χ,per_(LF) = 0.17.

The absorbance recovery of RF in starch after the second cycle of light exposure is shown by the dotted-line connected dots in the top part of Figure 4. The fast absorbance recovery component is more pronounced than after the first excitation, and the final absorbance is lower than reached in the first recovery. The same behavior was found for LF in starch as is shown by the dash-dotted line connected filled triangles in the top part of Figure 4. The second last filled triangle, and the second last dot were measured at relatively high relative humidity of ϕ_rh_ ≈ 0.66 (for preceding few days the ambient relative humidity varied between 50% and 69%). The last filled triangle and the last dot were measured after storing the samples for four days in a desiccator (ϕ_rh_ < 0.1) and relative humidity during measurement of ϕ_rh_ ≈ 0.45.

### 2.2. Fluorescence Photocycling

#### 2.2.1. Fluorescence Spectra and Fluorescence Quantum Yields

**Figure 5:**
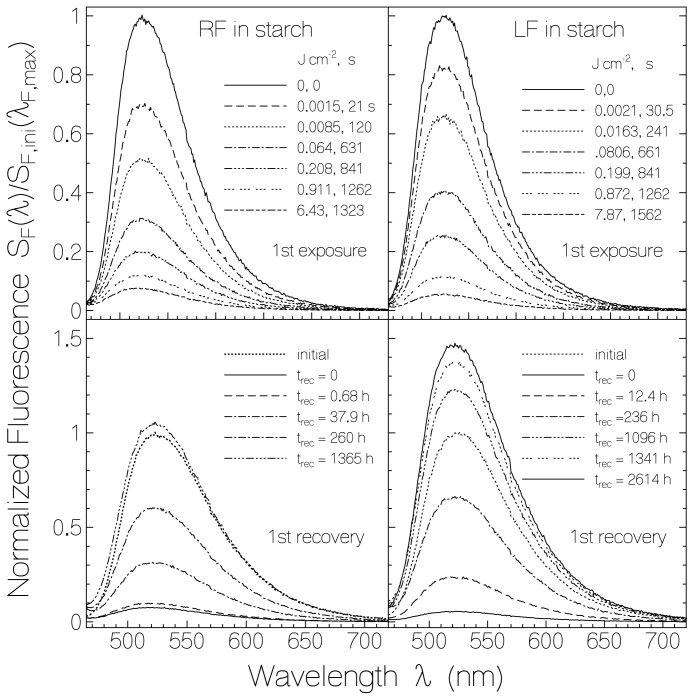
Fluorescence spectra of RF in starch (left part) and LF in starch (right part) belonging different exposed energy densities at λ_exc_ = 455 nm (top part) and different times of recovery after light switch-off.

The evolution of spectrally corrected fluorescence spectra of previously unexposed RF and LF in starch due to light exposure and dark recovery is shown in Figure 5 (light exposure at λ_exp_ = 455 nm, excitation wavelength for fluorescence detection λ_F,exc_ = 450 nm). The left figures belong to RF in starch and the right figures belong to LF in starch. The top parts show the light exposure situation, and the bottom parts show the situation of fluorescence recovery in the dark. The depicted fluorescence spectra are normalized to the initial fluorescence signal height at the wavelength of maximum fluorescence emission, *i.e.*, *S*_F_(λ)/*S*_F,ini_(λ_F,max_) is displayed (λ_F,max_(RF) = 523 nm, λ_F,max_(LF)= 525 nm). The shape of the fluorescence spectra is approximately independent of exposed light energy density and time of recovery. The fluorescence signal height decreased with light exposure starting already at very low light exposure. The fluorescence emission recovered after light exposure even beyond the original fluorescence signal height (see below).

**Figure 6:**
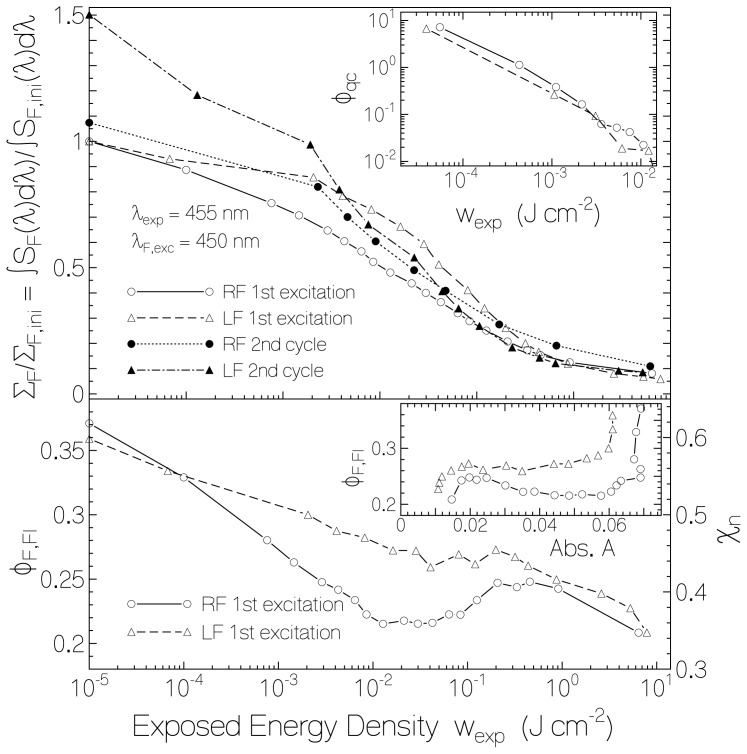
Top part: Dependence of normalized fluorescence emission of RF and LF in starch *versus* exposed energy density, *w*_exp_. Bottom part: Component specific fluorescence quantum yield, *φ_F,Fl_*, (left ordinate) and fraction of normal emitting flavin quinone, χ_n_, (right ordinate) *w*_exp_. Inset in top part: Quantum yield of photo-induced fluorescence quenching center formation, *φ_qc_*, *versus w*_exp_ for first excitation cycle. Inset in bottom part: Flavin quinone specific fluorescence quantum yield, *φ_F,Fl_*, *versus* corresponding absorbance, *A*, for first excitation cycle.

The dependence of the fluorescence efficiency on the exposed light energy density is shown in Figure 6. The solid line connected circles belong to RF in starch film, and the dashed line connected triangles belong to LF in starch film of the first excitation cycle. The top part of Figure 6 shows the decrease of the total (spectral integrated) fluorescence signal, ∑*_F_* = ∫ *S_F_* (λ)*d*λ, with exposed energy density *w*_exp_. The curves are normalized to the total initial fluorescence signal, ∑ *_F_*_,_*_ini_* = ∫ *S_F_*_,_*_ini_* (λ)*d*λ, (unexposed samples). Comparing the total fluorescence signal decrease with the absorbance decrease shown in Figure 3 one sees that the fluorescence emission decreased already at low exposed energy densities (*w*_exp_ < 10^−3^ J·cm^−2^) where the absorbance decrease was still negligible. For higher exposed energy densities (*w*_exp_ > 10^−3^ J·cm^−2^) the total fluorescence signal decreased in accordance with the reduction of flavin quinone to flavin hydroquinone (see Figure 3). It should be noticed that flavin semiquinone and flavin hydroquinone are non-fluorescent [2,39].

In the lower part of Figure 6 the component specific fluorescence quantum yield, *φ_F,Fl_*, [44] of riboflavin quinone and lumiflavin quinone in starch film *versus* exposed energy density (first excitation cycle) is plotted. The curves were obtained by normalizing the total fluorescence signals of the top part of Figure 6 to the absorbed fluorescence excitation light by flavin quinone and adjusting to the fluorescence quantum yield before light exposure. The fluorescence quantum yields before light exposure have been determined recently [27]. Their values are *φ_F,ini_*(RF/starch) = 0.37 and *φ_F,ini_* (LF/starch) = 0.36. The decrease of fluorescence quantum yield with light exposure indicates increased fluorescence quenching of flavin quinone in the course of exposure (photo-induced quenching center formation by starch—flavin restructuring). At the end of exposure the total fluorescence signal was stronger decreased than the absorbance or the fraction of retaining flavin quinone because the flavin quinone specific fluorescence quantum yield decreased with *w*_exp_.

The quantum efficiency, *φ_qc_*, of flavin-starch quenching center formation at low exposed energy density (*w*_exp_ < 10^−2^ J·cm^−2^) may be estimated from the decrease of fluorescence efficiency with absorbed excitation light. It is

(4) $$\varphi_{qc}=\frac{\Delta {\text{N}}_{qc}}{\Delta n_{ph,abs}}$$

where ΔN *_qc_* is the length-integrated number density increment of formed quenching centers, and Δ*n*_ph,abs_ is the corresponding number density of absorbed photons. At *w*_exp_, ΔN *_qc_* may be expressed by the amount of Fl component specific fluorescence quantum yield decrease Δ*φ_F,Fl_* according to

(5) $$\Delta {\text{N}}_{qc}(w_{\text{exp}})=\frac{\Delta \varphi_{F,Fl}(w_{\text{exp}})}{\varphi_{F,Fl,0}}{\text{N}}_{Fl}(w_{\text{exp}})=\frac{\Delta \varphi_{F,Fl}(w_{\text{exp}})}{\varphi_{F,Fl,0}}{\text{N}}_{Fl}(0)\chi_{Fl}(w_{\text{exp}})=\frac{\Delta \varphi_{F,Fl}(w_{\text{exp}})}{\varphi_{F,Fl,0}}\frac{A(0,\lambda )\text{ln}(10)}{\sigma_{a,Fl}(\lambda )}\chi_{Fl}(w_{\text{exp}})$$

The corresponding number density of absorbed photons at *w*_exp_ is

(6) $$\Delta n_{ph,abs}(w_{\text{exp}})=\frac{\Delta w_{\text{exp}}}{h\nu_{\text{exp}}}[1-T(\lambda_{\text{exp}},w_{\text{exp}})]\approx \frac{\Delta w_{\text{exp}}}{h\nu_{\text{exp}}}A(\lambda_{\text{exp}},w_{\text{exp}})\text{ln}(10)$$

The calculated curves of *φ_qc_* *versus w*_exp_ for RF and LF in starch are shown in the inset of the upper part of Figure 6. The quantum efficiency of flavin-starch quenching-center formation starts from *φ_qc_* ≈ 10 at low exposed energy density (*w*_exp_ ≤ 10^−5^ J cm^−2^) and decreases to *φ_qc_* ≈ 0.01 at *w*_exp_ ≈ 0.01 J cm^−2^. This result shows that initially one flavin molecule excitation caused a starch—flavin structure optimization (starch restructuring) which changed about 10 normal fluorescing flavin molecules, Fl_n_, in inert starch environment to non-fluorescent flavin molecules, Fl_qc_, in fluorescence quenching active starch environment (flavin–glucose quenching center formation). The total number density of flavin quinones is *N_Fl_* = *N_Fln_* + *N_Fl_qc__*. The quantum efficiency of flavin-starch quenching center formation decreased with increasing exposed energy density showing that the starch polymer rearrangement was getting more and more difficult. For *w*_exp_ > 10^−2^ J·cm^−2^ *N_qc_*(*w*_exp_)/*N_Fl_*(*w*_exp_) became rather constant and even decreased over a certain energy density range for RF in starch where *φ_F,Fl_* increased with *w*_exp_. The oxidized flavin specific fluorescence quantum yield *φ_F,Fl_* *versus* sample absorbance *A*(450 nm) is shown in the inset of the bottom part of Figure 6 for RF in starch (circles) and for LF in starch (triangles). The absorbance data belong to Figure 3. The decrease of fluorescence quantum yield without change of absorbance at the right side (beginning of light exposure) is clearly seen.

The dotted line connected dots in the top part of Figure 6 show the fluorescence behavior of RF in starch recovered in the dark at room temperature over a period of 4 month and re-exposed in a second cycle. The dash-dotted line connected filled triangles in the top part of Figure 6 show the fluorescence behavior of LF in starch recovered in the dark at room temperature over a period of 5 month and 20 days. For both RF and LF in starch the fluorescence quenching behavior was similar in the second excitation process as in the first excitation process.

**Figure 7:**
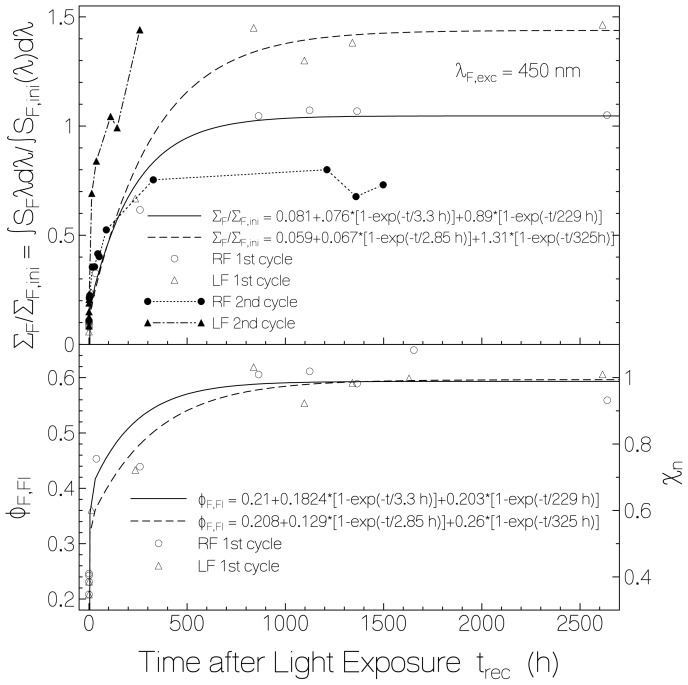
Top part: Dependence of normalized fluorescence emission *versus* time after end of exposure. Bottom part: Component specific fluorescence quantum yield of flavin quinone (left ordinate), and fraction of normal fluorescing flavin quinone (right ordinate) *versus* time after end of exposure.

The fluorescence recovery of RF and LF in starch film after light switch-off in the dark is shown in Figure 7. Circles and triangles belong to RF and LF after the first excitation process, respectively. In the top part the recovery of the total (spectral integrated) fluorescence signal (normalized to the initial total fluorescence signal), ∑_F_/∑_F,ini_, is depicted. The fluorescence recovery fits reasonably well to bi-exponential functions (solid curve for RF, dashed curve for LF) with the same recovery times, τ_rec,f_ and τ_rec,s_, as found for the absorbance recovery (Equation (3)). The total fluorescence signal of RF in starch recovered to approximately 1.05 times the initial fluorescence signal while the RF molecules re-oxidized only to a final fraction value of *χ*_RF,∞_ ≈ 0.67. The total fluorescence signal of LF in starch recovered to approximately 1.45 times the initial fluorescence signal while the LF molecules re-oxidized to a final fraction value of *χ*_LF,∞_ ≈ 0.85. This behavior indicates that the fluorescence quantum yield of the re-oxidized flavin is higher than the fluorescence quantum yield of the initial flavin.

The fluorescence recovery of RF in starch after a second cycle of light exposure is shown by the dotted-line connected dots in the top part of Figure 7. The situation of fluorescence recovery of LF in starch after second cycle of exposure is shown by the dash-dotted line connected filled triangles in the top part of Figure 7. In both cases the initial fast recovery after second light switch-off is more pronounced than after the first excitation. For RF in starch the final fluorescence recovery after the second excitation is lower than after the first excitation as is the absorbance recovery (dots in Figure 4). For LF in starch the final fluorescence recovery after the second exposure is about the same as after the first excitation. The second last filled triangle and the second last dot were measured at relatively high relative humidity of ϕ_rh_ ≈ 0.66 whereby the humidity in the laboratory was in the range between 50% and 69% for a few days. The last filled triangle and the last dot were measured after storing the samples for four days in a desiccator (ϕ_rh_ < 0.1) before measuring (relative humidity in laboratory during measurement was ϕ_rh_ ≈ 0.45).

The dependence of the component specific fluorescence quantum yield, *φ_F,Fl_*, of flavin quinone on the dark time after first light exposure is depicted in the lower part of Figure 7. For both, RF and LF in starch, the fluorescence quantum yield at the end of light exposure was *φ_F,Fl_*(0) ≈ 0.21, and it increased to *φ_F,Fl_*(∞) ≈ 0.60. The initial fluorescence quantum yields were *φ_F,RF,ini_* = 0.37 and *φ_F,LF,ini_* = 0.36 [27]. The increase of fluorescence quantum yield after recovery beyond the initial values indicates that already before light exposure flavin—starch quenching centers were present which lowered the initial light emission. Obviously, the thermal sample recovery in the dark period did not create remarkably flavin-starch quenching centers along with the flavin re-oxidation. Assuming that no flavin-starch quenching centers were left after saturating light exposure and complete recovery, the fraction of initially present flavin-strach quenching centers is estimated to be *χ_qc,ini_* ≈ (*φ_F,Fl,recovered_* − *φ_F,Fl,ini_*)/*φ_F,Fl,recovered_* giving *χ*_qc,ini_(RF) ≈ 0.38 and *χ*_qc,ini_(LF) ≈ 0.4. The fraction of flavin-starch quenching centers at the end of first light exposure is similarly estimated to be *χ*_qc,end_ ≈ (*φ_F,Fl,recoverred_* − *φ_F,Fl,end_*)/*φ_F,Fl,recovered_* giving χ_qc,end_ ≈ 0.65 for both RF quinone and LF quinone (*φ_F,Fl,end_* ≈ 0.21 in both cases).

The fraction of normal emitting flavin quinone molecules is *χ_n_* = 1 − *χ_qc_* ≈ *φ_F,Fl_*/*φ_F,Fl,recovered_*. The curves in the lower part of Figure 6 with the right ordinate give the dependence of χ_n_ *versus* exposed energy density. The curves in the lower part of Figure 7 with the right ordinate give the development of χ_n_ with recovery time after light switch-off.

#### 2.2.2. Fluorescence Lifetimes

The fluorescence lifetimes of RF in starch film were measured before light exposure, during light exposure, and after dark recovery (first excitation cycle) with a mode-locked laser system and a micro-channel-plate photomultiplier (MCP) oscilloscope system (time resolution *t*_res_ ≈ 500 ps). The fluorescence signals fitted well to a single exponential decay. The fluorescence lifetime did not change with exposed light energy density and did not change during dark recovery. The measured 1/e-lifetime was τ_F,RF_ = 4.32 ± 0.03 ns. For LF in starch the fluorescence lifetime was measured before light exposure and after dark recovery (first excitation cycle). In both cases the same fluorescence lifetime of τ_F,LF_ = 4.57 ± 0.07 ns was measured.

Additionally, the time-resolved fluorescence of LF in starch film was measured before light exposure and after dark recovery with the mode-locked laser system and an ultrafast streak-camera (time resolution *t*_res_ ≈ 10 ps). For the unexposed sample the rise of the fluorescence signal was steeper than for the sample after complete dark recovery (4 month of dark recovery). This finding indicates the presence of an unresolved fast decaying fluorescence component for the unexposed sample. A convolution analysis [36] using *S_F_* (*t*) = ∫ *g* (*t*′)*S_F_*_,δ_ (*t* − *t*′)*dt*′ with Gaussian response function *g*(*t*) =π ^−1/2^ exp(−*t*^2^/*t_res_*^2^) gave a single exponential fluorescence decay for the dark recovered sample according to *S_F_*_,δ_ (*t*) = *S_F_*_,0_*θ* (*t*) exp(−*t* /τ*_F_*_,_*_n_*) where τ_F,_*_n_* is the fluorescence lifetime determined by the MCP measurements (τ_F,n_ = τ_F_ = 4.57 ns). θ(*t*) is the Heaviside step-function (θ(*t*)= 0 for *t* < 0 and θ(*t*) = 1 for for *t* ≥ 0). For the unexposed LF sample the measured traces fitted well to bi-exponential fluorescence decay according to *S* *_F_*_,δ_ (*t*) = *S* *_F_*_,0_*θ*(*t*)[χ *_n_* exp( −*t* /τ*_F_*_,_*_n_*) + χ *_qc_* exp(−*t* /τ *_F_*_,_*_qc_*)] with *χ*_n_ = 0.6, τ_F,_*_n_* = 4.57 ns, χ*_qc_* = 1 − χ*_n_* = 0.4, and τ_F,qc_ = 1.5 ± 0.5 ps.

The experimental situation of fluorescence quantum yield decrease and constant fluorescence lifetime with exposed energy density at moderate time resolution is typical for static fluorescence quenching with increasing quenching complex concentration *χ*_qc_ where the short fluorescence lifetime τ_F,qc_ of the emitter-quencher-complexes cannot be resolved [32–35]. The streak-camera traces convolution analysis revealed a fluorescence lifetime of the flavin-starch quenching centers of τ_F,qc_ = 1.5 ± 0.5 ps.

The static fluorescence quenching observed here has some similarity with static fluorescence of FAD in neutral aqueous solution (see [2,37,45,46] and references therein) where for part of the FAD molecules the isoalloxazine moiety and the adenine moiety are un-stacked (normal fluorescing) and for the other part of the FAD molecules the isoalloxazine moiety and the adenine moiety are stacked (very weakly fluorescent due to efficient electron transfer between isoalloxaine and adenine and subsequent charge recombination).

### 2.3. Delayed Luminescence Studies

**Figure 8:**
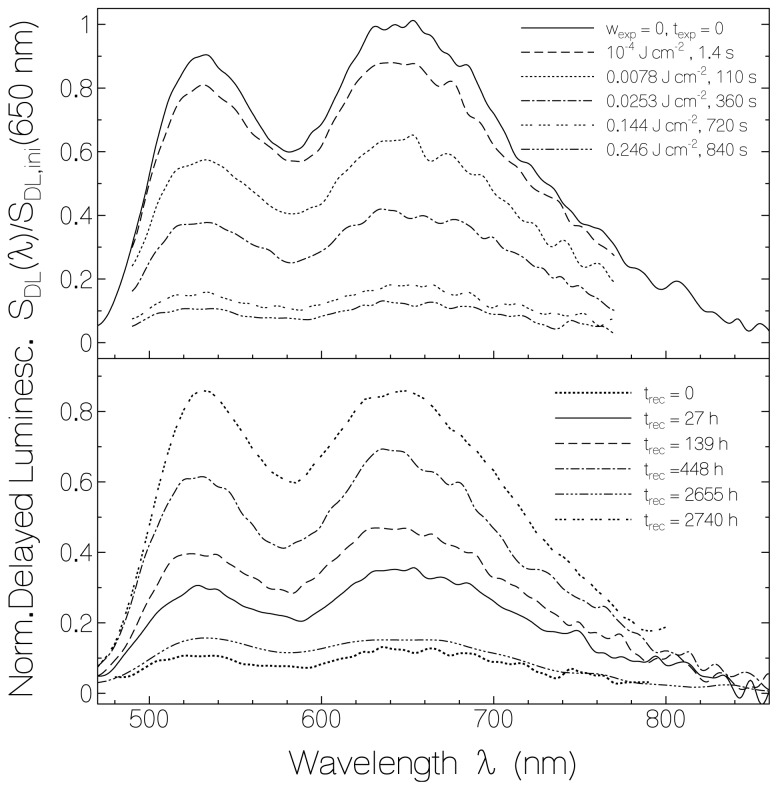
Delayed luminescence spectra of riboflavin in starch film (first excitation cycle). Top part: Dependence on exposed light energy density at λ_exp_ = 455 nm. Bottom part: Dependence on storage time in the dark after light exposure. Curve belonging to *t*_rec_ = 2655 h: sample was measured at ϕ_rh_ ≈ 0.66 after a few humid days ( varied between 0.50 and 0.69). Curve belonging to *t*_rec_ = 2744 h: sample was measured after 4 days stored in a desiccator.

**Figure 9:**
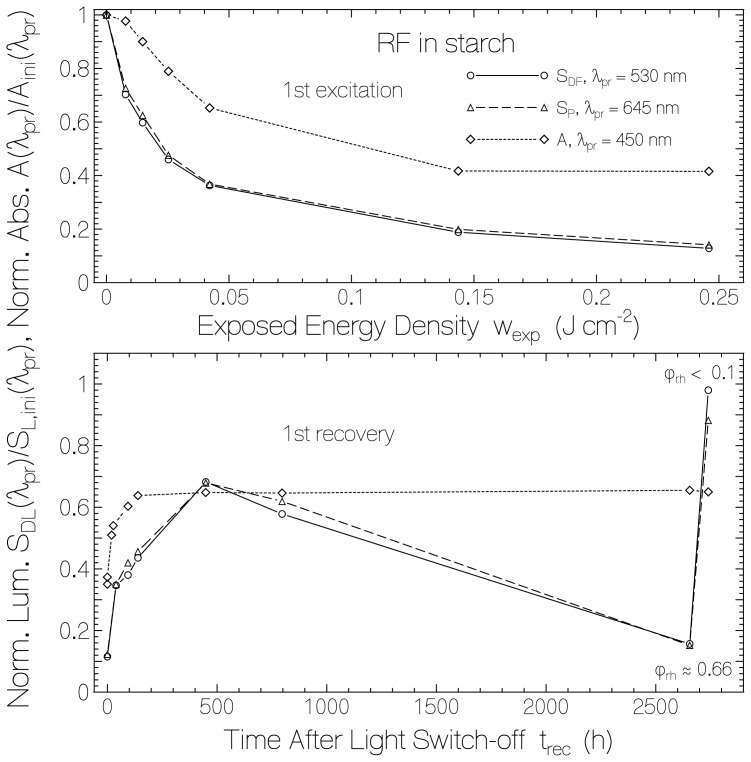
Dependence of normalized delayed fluorescence (*S*_DF_/*S*_DF,ini_) at λ_pr_ = 530 nm, phosphorescence (*S*_P_/*S*_P,ini_) at 645 nm, and absorbance (*A*/*A*_ini_) at 450 nm of riboflavin in starch *versus* exposed energy density at 455 nm (top part) and *versus* recovery time after end of exposure (bottom part).

**Figure 10:**
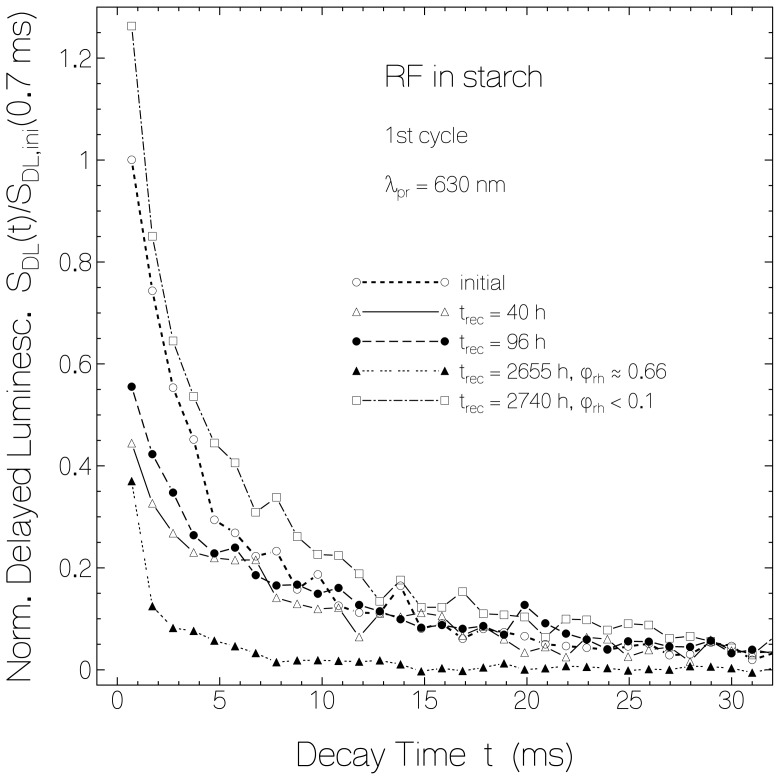
Delayed luminescence signal decay of RF in starch before and after first sample exposure (parameters of Figures 1, 8 and 9 apply).

In the photo-reduction studies on RF and LF in starch films also the delayed luminescence DL (phosphorescence P and delayed fluorescence DF) behavior was investigated. Delayed luminescence spectra and signal decay traces were measured. RF in starch and LF in starch behaved similar. Below in Figures 8–10 only results are shown for RF in starch obtained for the first excitation—recovery cycle. The delayed luminescence behavior of the samples before light exposure was reported in [27].

In Figure 8 spectrally corrected delayed luminescence spectra are shown for RF in starch (same sample as used for Figure 1, fluorimeter gate opening 200 μs after sample excitation with 2 μs pulses, gate width 5 ms). The top part shows spectra belonging to light exposure at λ_exp_ = 455 nm with different exposed energy densities, and the bottom part shows spectra belonging to different times of recovery in the dark. The spectra are normalized to the initial luminescence signal at 650 nm, *i.e.*, *S*_DL_(λ)/*S*_DL,ini_(650 nm) is presented. The emission band peaking around 650 nm belongs to phosphorescence, and the emission band peaking around 530 nm belongs to delayed fluorescence (for details see [27]). The spectral shapes retain approximately unchanged during light exposure and during recovery in the dark. The luminescence signal heights decreased with light exposure and increased after light switch-off in the dark. The curve belonging to for *t*_rec_ = 2655 h was measured at ϕ_rh_ = 0.66 after a few days of high humidity in the range of 50% to 69% in the laboratory. The strong reduction of delayed luminescence at high relative humidity is clearly seen. Above ϕ*_rh_* = 50% the starch films begin to become slightly permeable to oxygen [47] and the water vapor permeability increases [48] with softening of the films. The phosphorescence is thought to be decreased because of flavin triplet deactivation by adjacent molecular oxygen according to [49,50] _3_Fl_n_^*^ + ^3^O_2_ →^1^ Fl_n_ + ^1^O_2_^*^. The curve belonging to *t*_rec_ = 2740 h was measured at ϕ_rh_ = 0.45 after storing the sample for four days in a desiccator (ϕ_rh_ < 0.1). The delayed luminescence spectrum recovered nearly to the initial spectrum. The delayed fluorescence peak became similar high as the phosphorescence peak (some enhancement of delayed fluorescence).

The development of the delayed luminescence at λ_pr_ = 530 nm (dominant delayed fluorescence, line connected circles) and at λ_pr_ = 645 nm (dominant phosphorescence, dashed line connected triangles) *versus* exposed energy density at λ_exp_ = 455 nm and *versus* recovery time in the dark is shown in Figure 9 for RF in starch film (data taken from Figure 8). The curves are normalized to the initial delayed luminescence signal heights at the same probe wavelengths. For comparison the normalized absorbance dependence *A*(450 nm)/*A*_ini_(450 nm) on exposed energy density and on time after exposure is included (dotted line connected diamonds). The delayed luminescence signal followed the exposure dependence and the recovery dependence of the fluorescence signal. For the second last data points in the bottom part of Figure 9 the luminescence signal was small because of the high relative humidity situation. After storing the sample in a desiccator (last data points in the bottom part of Figure 9) the delayed luminescence signal approached the initial delayed luminescence signal while the absorbance recovered only to about 65%. The final low-humidity flavin quinone specific delayed luminescence quantum yield, *φ_DL,Fl_*, reached higher values than the initial unexposed sample. This behavior is the same as was found for the fluorescence. Both the fluorescence and the low-humidity delayed luminescence efficiency is proportional to the mole fraction of normal fluorescing flavin quinone χ_n_.

In Figure 10 the time-resolved luminescence signal decay for RF in starch before light exposure and for certain times after first exposure is shown (same sample used as for Figures 1, 8 and 9). The curves are determined by the mole-fraction χ_n_ of normal fluorescing flavin quinone and the dissolved molecular oxygen dependent delayed luminescence lifetime shortening. The curve measured at *t*_rec_ = 2655 h under high relative humidity conditions shows a strong phosphorescence lifetime shortening. For the other curves the temporal decay curves have a similar shape. The curve measured at *t*_rec_ = 2740 h under low relative humidity conditions gave slightly higher luminescence signal than the initial unexposed sample.

The data analysis of unexposed RF and LF in starch films was carried out in [27] assuming the presence of only normal fluorescing flavins Fl_n_. The presence of flavins Fl_qc_ in quenching centers changes the radiative S_1_-state lifetime (Equation (2) in [27]) to τ*_rad_*_,_*_S_*__1__ =τ*_F_*_,_*_n_*/*φ_F_*_,_*_n_* (*φ_F,n_* ≈ 0.60). The inclusion of the static fluorescence quenching causes the correction of a few parameters for RF in starch and LF in starch to: τ*_rad_*_,_*_S_*__1__ (RF) = 7.2 ns, σ̄*_a_*, *_S_*__0_−_*_S_*__1__ (RF) = 1.34 × 10^−17^ cm^2^, σ_a,max_(RF) = 7.2 × 10^−17^ cm^2^, τ_F_(LF) = 4.57 ns (new measurement because of some degradation of previous sample), τ*_rad_*_,_*_S_*__1__ (LF) = 7.6 ns, σ̄*_a_*, *_S_*__0_−_*_S_*__1__ (LF) = 1.28 × 10^−17^ cm^2^, σ_a,max_(LF) = 6.85 × 10^−17^ cm^2^, *φ_ISC_*(LF) = 0.38, *k*_ISC_(LF) = 8.32 × 10^7^ s^−1^, and *k_T_*__1_,_*_S_*__1__ (LF) = 1.13 s^−1^.

### 2.4. Discussion of Reduction—Oxidation Photocycle Dynamics

**Figure 11:**
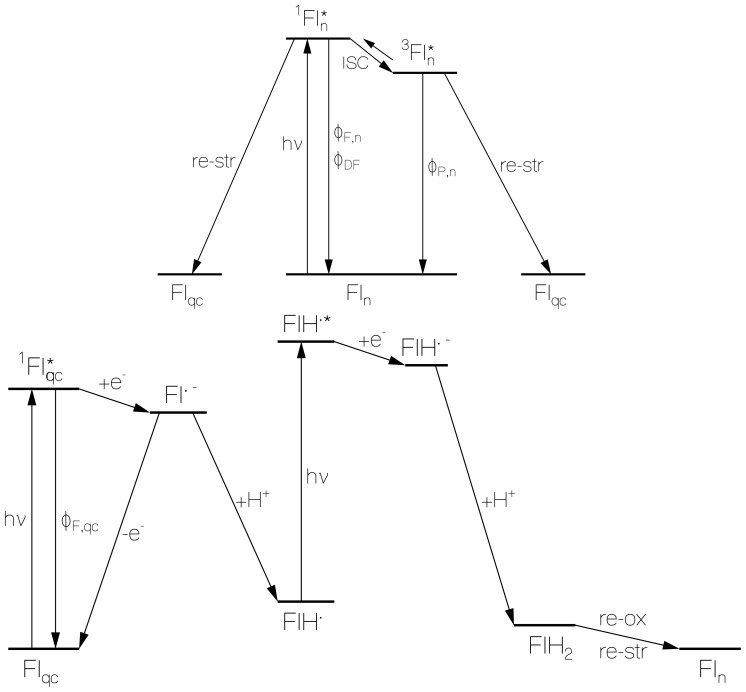
**Top part**: Scheme of normal flavin (Fl_n_) excitation leading to flavin-starch quenching center formation (Fl_qc_) due to starch restructuring. **Bottom part**: Fl_qc_ excitation causing flavin semiquinone FlH^·^ formation by electron and proton transfer; photo-excitation of FlH^·^ causing flavin hydroquinone FlH_2_ formation by electron and proton transfer; and thermal FlH_2_ re-oxidation with starch restructuring to normal fluorescing flavin quinone Fl_n_. The photo-excitation dynamics of Fl_qc_ is illustrated in the lower part of Figure 11.

The flavin behavior in starch may be described by a two-component system consisting of normal fluorescing flavins Fl_n_ and flavins Fl_qc_ forming flavin-glucose fluorescence quenching centers. The proposed photocyclic dynamics and inter-conversion of Fl_n_ and Fl_qc_ is illustrated in Figure 11.

The formation of flavin-starch fluorescence quenching centers Fl_qc_ by photo-excitation of normal fluorescing flavin quinone Fl_n_ is illustrated in the top part of Figure 11. Photo-excitation of Fl_n_ leads to radiative relaxation, internal conversion, intersystem-crossing, back-intersystem-crossing (delayed fluorescence), radiative triplet-singlet relaxation (phosphorescence), and non-radiative triplet relaxation to the singlet ground-state. The energy released by non-radiative decay and excited-state charge distribution differences compared to the ground-state are thought to cause starch re-conformation to flavin-glucose fluorescence quenching center formation (Fl_qc_) on the time range of phosphorescence decay (10 ms time scale). It should be noted that in the prepared flavin doped starch samples there were already fluorescence quenching centers present before light exposure.

The photo-excitation of Fl_qc_ is thought to cause a reductive electron transfer [35,51] from properly arranged glucose G(OH)_2_ to flavin Fl according to

(R1) $$\text{Fl}+\text{G}{\left(\text{OH}\right)}_{2}\overset{\text{hv}}{\to }\text{Fl}*+\text{G}{\left(\text{OH}\right)}_{2}\overset{{\text{e}}^{-}}{\to }{\text{Fl}}^{•-}+\text{G}{{\left(\text{OH}\right)}_{2}}^{•+}$$

whereby flavin quinone radical anion Fl·^−^ and glucose radical cation G(OH)_2_·^+^ is formed. The electron transfer quenches the fluorescence (time constant of electron transfer is τ_F,qc_ ≈ 1.5 ps). Fl^·−^ dominantly relaxes back to Fl_qc_ by charge recombination. Part of Fl^·−^ converts to neutral flavin semiquinone FlH^·^ by proton transfer (quantum efficiency ≥ *φ_red_*) according to

(R2) $${\text{FL}}^{•-}+\text{G}{{\left(\text{OH}\right)}_{2}}^{•+}\overset{{\text{H}}^{+}}{\to }{\text{FLH}}^{•}+{\text{GO}}_{2}{\text{H}}^{•}$$

The incident light excites FlH^·^ to FlH^·^* and causes subsequent electron transfer to flavin semiquinone radical anion according to

(R3) $${\text{FIH}}^{•}+{\text{GO}}_{2}{\text{H}}^{•}\overset{\text{hv}}{\to }{\text{FIH}}^{•}*+{\text{GO}}_{2}{\text{H}}^{•}\overset{{\text{e}}^{-}}{\to }{\text{FIH}}^{•-}+{\text{GO}}_{2}{\text{H}}^{•+}$$

A second proton transfer from the single oxidized glucose radical cation to the flavin semiqinone radical anion leads to flavin hydroquinone and oxy-glucose (oxystarch) according to

(R4) $${\text{FIH}}^{•-}+{\text{GO}}_{2}{\text{H}}^{•+}\overset{{\text{H}}^{+}}{\to }{\text{FIH}}_{2}+{\text{GO}}_{2}$$

The total quantum efficiency of FlH_2_ formation is *φ_red_*. In the dark there occurs thermal flavin hydroquinone re-oxidation to flavin quinone by oxystarch reduction to normal starch and a starch restructuring.

**Scheme 1:**
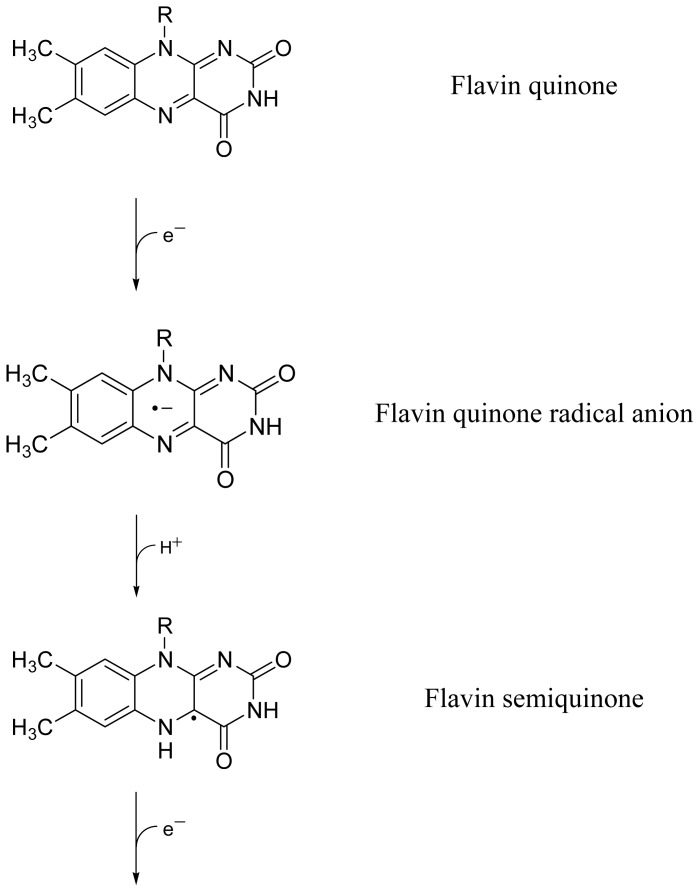

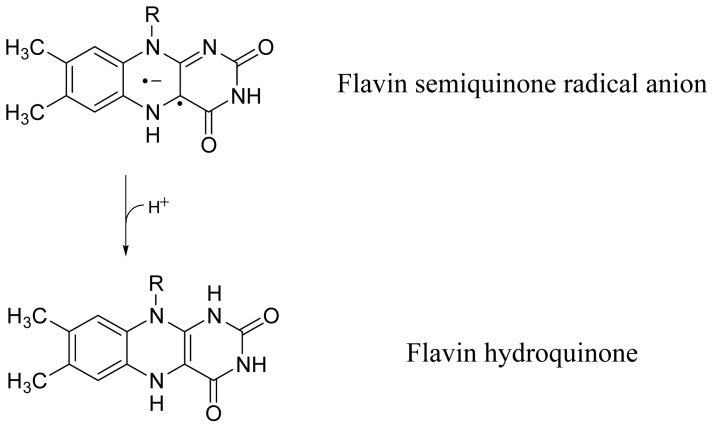
Reduction of fully oxidized flavin to fully reduced flavin via semi-reduced flavin in two electron and proton transfer steps.

**Scheme 2:**
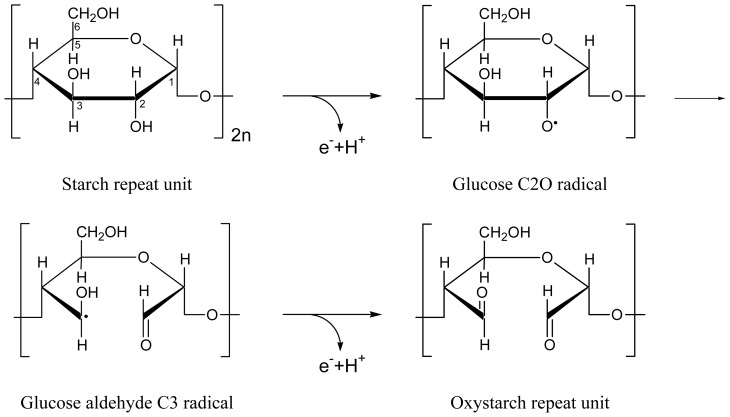
Oxidation of a glucose repeat unit of starch to a di-aldehyde glucose repeat unit of oxystarch in two steps of electron and proton transfer.

The flavin reduction from Fl to FlH_2_ is visualized in Scheme 1. The corresponding partial starch oxidation to oxystarch where glucose repeat units are oxidized to oxy-glucose repeat units is illustrated in Scheme 2. Information on photochemical degradation of starch to oxystarch (di-aldehyde formation) is found in [28–31].

The starch oxidation to oxystarch during light exposure and the oxystarch reduction to starch after light switch-off is concluded form the fact that the flavin reduction and subsequent re-oxidation is repeatable. For LF in starch no photo-induced intra-molecular reduction is possible, the oxidation of starch is necessary for the process to occur. Re-oxidation of LFH_2_ to LF might be caused by oxygen [52] dissolved in starch and penetrating into starch, but without oxystarch back-reduction to starch a repetition of cyclic photo-reduction and re-oxidation would not be possible. For RF in starch photo-induced intra-molecular reduction is possible by hydrogen abstraction from the ribityl part [5]. This process is thought to take place additionally to the starch oxidation since the quantum efficiency of photo-reduction is higher for RF in starch than for LF in starch, and the RFH_2_ back re-oxidation in the dark is less complete than for LFH_2_ (no back reduction of the oxidized ribityl part is indicated). In the starch film forming process by solution heating [27] the samples get partly degassed (oxygen solubility in solution decreases with rising temperature). Starch films in dry air (*ϕ_rh_* < 50%) are air impermeable (see results in [47,48] for free standing starch films of 20 μm thickness, our estimated film thickness is ≈ 36 μm). Photo-excitation studies on lumiflavin doped polystyrene films which are air permeable [53,54] did not show flavin photo-reduction (own unpublished results).

### 2.5. Comparison with Photocycling of Natural Flavin based Blue-Light Photoreceptors

The found artificial photocycle behavior of photo-induced reduction and dark re-oxidation of flavins in starch films resembles the photocycle behavior of natural flavin-based blue-light photoreceptors.

In LOV domains of phototropin charge-transfer reactions between photo-excited singlet and triplet states of FMN and the protein (cystein residue) are involved in the meta-stable FMN-C4a-cysteinyl adduct formation [18,55]. The FMN-C4a-cysteinyl adduct recovers thermally back to FMN and cystein residue typically on a minute timescale [12–15,18,55].

In BLUF proteins [14,16,19,56] and BLUF domain containing photo-activated enzymes [20,57,58] photo-excitation of FAD or FMN causes tyrosine to flavin quinone electron transfer forming FAD^·−^ or FMN^·−^ with protein-flavin hydrogen-bond reorganization during the sub-nanosecond Fl^·−^ charge-recombination lifetime giving the signaling state (light-adapted state) for effector domain activation [14,19,20,57,59]. In light adapted BLUF domains flavin reduction to flavin semiquinone FlH^·^ and anionic flavin hydroquinone FlH^−^ may take place with partial re-oxidation after light switch-off [20,57,59]. In the dark a hydrogen-bond back arrangement to the original situation takes place on a second to minute timescale [14,16,19,20,56–59].

In cryptochromes the primary receptor state—signaling state photocycle is given by photo-induced Tyr or Trp electron transfer to FAD forming FAD·^−^ which is stabilized over a time range of seconds by positive charge separation along a Trp triade [17,24,25,60]. FAD^·−^ may further reduce to FADH^·^ and FADH^−^ [25,60,61]. The reduced FAD may partially re-oxidize in the dark on a minute to hour time scale [25,61].

### 2.6. Comparison with Dye Reduction and Re-Oxidation by Agents

The photo-induced formation of reversible dye radicals in the triplet state by application of reducing agents in aerobic liquid solutions was reported to be a frequently occurring phenomenon [62]. It was observed in rhodamine, oxazine, thiazine, and cyanine dyes in the presence of reducing agents like β-mercaptoethylamine or glutathione [62]. Photo-excitation caused dye reduction (radical formation) in the triplet state, and the speed of re-oxidation in the dark was determined by the dissolved oxygen content. The photo-induced formation of reversible dye radicals plays a crucial role in live-cell super-resolution imaging fluorescence microscopy [62,63]. Flavin photo-reduction in liquid solutions containing reducing agents and oxygen dependent re-oxidation was investigated in [6,7,43,64].

In the flavin doped solid starch films studied here, the starch host acts as reducing agent for photo-excited flavin. The thereby generated oxystarch acts as oxidizing agent for reduced flavin in its ground-state. In the case of riboflavin in starch hydrogen abstraction from the ribityl chain [5,43,65–68] is thought to contribute to the isoalloxazine core reduction and is thought to be responsible for the incomplete re-oxidation in the dark. Dissolved oxygen is generally responsible for reduced flavin re-oxidation [2–7]. Starch films at low relative humidity (ϕ_rh_ < 0.5) are impermeable to oxygen and the retained oxygen in starch is immobile. Therefore flavin re-oxidation by oxygen in starch under dry ambient conditions is thought to be of minor importance.

## 3. Experimental Section

Riboflavin (molar mass *M*_RF_ = 376.36 g·mol^−1^), lumiflavin (*M*_LF_ = 256.3 g·mol^−1^), and starch (from potatoes, treated with glycerol at 190 °C according to Zulkowsky [69], repeat unit: C_6_O_5_H_10_, molar mass of repeat unit 162 g·mol^−1^, mass density ρ_ST_ ≈ 1.55 g·cm^−3^) were purchased from Sigma-Aldrich and used as delivered. The solvent water was de-ionized in a Millipore water purifier and used in this form. The starch films were prepared from flavin-water and starch-water solutions on fused silica plates (25 mm diameter, 3 mm thickness). The thickness of the starch films was approximately 36 μm. The flavin concentration in the starch films was roughly 8 × 10^−3^ mol·dm^−3^. Details of sample preparation are reported in [27]. All measurements were carried out at room temperature (20 °C–23 °C) under ambient conditions and the samples were stored in the dark at room temperature under ambient conditions. Except stated different, the ambient relative humidity was low in the range between 20% and 50%.

For the photo-reduction of flavin doped starch films the samples were excited at 455 nm with a LED light source (LEDC1 from Thorlabs). The excitation light intensity was varied with optical neutral density filters (from Schott). The excitation intensity was measured with a power meter (Model PD 300-UV-SH photodiode detector head with NOVA power monitor from Ophir).

The absorption spectra were measured with a spectrophotometer (Cary 50 from Varian). The fluorescence spectra, delayed luminescence spectra, and delayed luminescence lifetimes were measured with a fluorimeter (Cary Eclipse from Varian). Experimental details are found in [27].

Fluorescence lifetimes of the samples were measured using a mode-locked titanium-sapphire laser oscillator amplifier system (Hurricane from Spectra-Physics) for sample excitation (second harmonic pulses of 3 ps duration, wavelength 400 nm, and energy ≈ 100 μJ). For fluorescence detection in most cases a micro-channel-plate photomultiplier (type R1564U-01 from Hamamatsu) was used in connection with a fast digital oscilloscope (LeCroy 9362) giving a time resolution of *t*_res_ ≈ 500 ps. The fluorescence emission in front-face collection arrangement was directed to the detector via a broadband interference filter (transmission range 500 nm–600 nm) and a magic angle polarizer (excitation light was vertically polarized, the polarizer in the detection path was oriented at an angle of 54.7° to the vertical). For higher fluorescence signal time resolution (*t*_res_ ≈ 10 ps) a streak camera (type C1587 temporal disperser with M1952 high-speed streak unit from Hamamatsu) was used [70].

## 4. Conclusions

Photo-excitation of the flavins riboflavin and lumiflavin in solid starch films caused repeatable reversible reduction (photo-induced conversion of oxidized flavin to fully reduced flavin via semi-reduced flavin and partial bi-exponential re-oxidation in the dark). The flavin doped starch films may be considered as artificial flavin based blue-light photoreceptors since they exhibit a similar flavin reduction—re-oxidation photocycle dynamics as the natural biological blue-light photoreceptors (LOV domains, BLUF proteins, cryptochromes). The role of the amino acid residues Cys, Tyr and Trp in the proteins of natural flavin-based blue-light photoreceptors is taken over in the artificial flavin doped starch films by the reducing action of the glucose repeat units of starch on photo-excited flavins and by the oxidizing action of di-aldehyde groups in the formed oxystarch on ground-state flavin in the reduced form.

A quantitative analysis of the photo-reduction dynamics of riboflavin and lumiflavin in starch was carried out determining the excitation energy density dependent quantum efficiency of flavin-starch photo-reduction center formation, *φ_qc_*, and the excitation energy density dependent quantum yield of flavin hydroquinone formation, *φ_red_*. Similar photo-reduction and re-oxidation behavior is expected for FMN and FAD in starch.

## References

[1] Friedrich W (1988). Vitamins.

[2] Müller F (1991). Chemistry and Biochemistry of Flavoenzymes.

[3] Heelis F. (1982). The photophysical and photochemical properties of flavins (isoalloxazines). Chem. Soc. Rev.

[4] Silva E., Edwards A.M. (2006). Flavins: Photochemistry and Photobiology; Comprehensive Series in Photochemistry and Photobiology.

[5] Moore W.M., Ireton R.C. (1977). The photochemistry of riboflavin—V. The photodegradation of isoalloxazines in alcoholic solvents. Photochem. Photobiol.

[6] Görner H. (2007). Oxygen uptake after electron transfer from amines, amino acids and ascorbic acid to triplet flavins in air-saturated aqueous solution. J. Photochem. Photobiol. B.

[7] Zhang Y., Görner H. (2009). Flavin-sensitized photo-oxidation of lysozyme and serum albumin. Photochem. Photobiol.

[8] Green M., Tollin G. (1968). Flash photoysis of flavins. I. Photoreduction in non-aqueous solvents. Photochem. Photobiol.

[9] Grodowski M.S., Veyret B., Weiss K. (1977). Photochemistry of flavins. II. Photophysical properties of alloxazines and isoalloxazines. Photochem. Photobiol.

[10] Lasser N., Feitelson J. (1975). Excited-state reactions of oxidized flavin derivatives. Photochem. Photobiol.

[11] Hemmerich P., Knappe W.R., Kramer H.E.A., Traber R. (1980). Distinction of 2e^−^ and 1e^−^ reduction modes of the flavin chromophore as studied by flash photolysis. Eur. J. Biochem.

[12] Batschauer A (2003). Photoreceptors and Light Signalling; Comprehensive Series in Photochemistry and Photobiology.

[13] Briggs W.R., Spudich J.L. (2005). Handbook of Photosensory Receptors.

[14] Losi A. (2007). Flavin-based blue-light photoreceptors: A photobiophysics update. Photochem. Photobiol.

[15] Briggs W.R., Huala E. (1999). Blue-light photoreceptors in higher plants. Annu. Rev. Cell Dev. Biol.

[16] Gomelsky M., Klug G. (2002). BLUF: A novel FAD-binding domain involved in sensory transduction in microorganisms. Trends Biochem. Sci.

[17] Sancar A. (2003). Structure and function of DNA photolyase and cryptochrome blue-light photorecetors. Chem. Rev.

[18] Holzer W., Penzkofer A., Hegemann P. (2005). Photophysical and photochemical excitation and relaxation dynamics of LOV domains of Phot from *Chlamydomonas reinhardtii*. J. Lumin.

[19] Zirak P., Penzkofer A., Hegemann P., Mathes T. (2007). Photo dynamics of BLUF domain mutant H44R of AppA from *Rhodobacter sphaeroides*. Chem. Phys.

[20] Tyagi A., Penzkofer A., Griese J., Schlichting I., Kirienko N.V., Gomelsky M. (2008). Photodynamics of blue-light-regulated phosphodiesterase BlrP1 protein from *Klebsiella pneumonia* and its photoreceptor BLUF domain. Chem. Phys.

[21] Bouly J.-B., Schleicher E., Sese M.D., Vandenbussche F., van der Straeten D., Bakrim N., Meier S., Batschauer A., Galland P., Bittl R. (2007). Cryptochrome blue light photoreceptors are activated through interconversion of flavin redox states. J. Biol. Chem.

[22] Banerjee R., Schleicher E., Meier S., Viana R.M., Pokorny R., Ahmad M., Bittl R., Batschauer A. (2007). The signaling state of *Arabidopsis* cryptochrome 2 contains flavin semiquinone. J. Biol. Chem.

[23] Zikihara K., Ishikawa T., Todo T., Tokutomi S. (2008). Involvement of electron transfer in the photoreaction of zebrafish cryptochrome-dash. Photochem. Photobiol.

[24] Song S.-H., Öztürk N., Denaro T.R., Arat N.Ö., Kao Y.-T., Zhu H., Zhong D., Reppert S.M., Sancar A. (2007). Formation and function of flavin anion radical in cryptochrome 1 blue-light photoreceptor of monarch butterfly. J. Biol. Chem.

[25] Zirak P., Penzkofer A., Moldt J., Pokorny R., Batschauer A., Essen L.-O. (2009). Photocycle dynamics of the E149A mutant of cryptochrome 3 from *Arabidopsis thaliana*. J. Photochem. Photobiol. B.

[26] Immeln D., Pokorny R., Herman E., Moldt J., Batschauer A., Kottke T. (2010). Photoreaction of plant and DASH cryptochromes probed by infrared spectroscopy: The neutral radical state of flavoproteins. J. Phys. Chem. B.

[27] Penzkofer A. (2012). Photoluminescence behavior of riboflavin and lumiflavin in liquid solutions and solid films. Chem. Phys.

[28] Peat S., Bourne E.J., Whelan W.J. (1948). Photochemical degradation of starch. Nature.

[29] Hofreiter B.T., Alexander B.H., Wolff I.A. (1955). Rapid estimation of dialdehyde content of periodate oxystarch through quantitative alkali consumption. Anal. Chem.

[30] Phillips G.O., Rickards T (1969). Photodegradation of carbohydrates. Part IV. Direct photolysis of d-glucose in aqueous solution. J. Chem. Soc. B.

[31] Bertolini A.C., Mestres C., Raffi J., Buléon A., Lerner D., Colonna P. (2001). Photodegradation of cassava and corn starches. J. Agric. Food Chem.

[32] Drössler P., Holzer W., Penzkofer A., Hegemann P. (2003). Fluorescence quenching of aqueous solutions of riboflavin by methionin and cystein. Chem. Phys.

[33] Förster T (1951). Fluoreszenz Organischer Verbindungen.

[34] Lakowicz J.R. (2006). Principles of Fluorescence Spectroscopy.

[35] Valeur B (2002). Molecular Fluorescence. Principles and Applications.

[36] Drössler P., Holzer W., Penzkofer A., Hegemann P. (2002). pH dependence of the absorption and emission behaviour of riboflavin in aqueous solution. Chem. Phys.

[37] Islam S.D.M., Susdorf T., Penzkofer A., Hegemann P. (2003). Fluorescence quenching of flavin adenine dinucleotide in aqueous solution by pH dependent isomerisation and photo-induced electron transfer. Chem. Phys.

[38] Tyagi A., Penzkofer A. (2010). pH dependence of the absorption and emission behaviour of lumiflavin in aqueous solution. J. Photochem. Photobiol. A.

[39] Song S.-H., Dick B., Penzkofer A., Hegemann P. (2007). Photo-reduction of flavin mononucleotide to semiquinone form in LOV domain mutants of blue-light receptor Phot from *Chlamydomonas reinhardtii*. J. Photochem. Photobiol. B.

[40] Edmondson D.E., Tollin G. (1983). Semiquinone formation in flavo- and metalloflavoproteins. Top. Curr. Chem.

[41] Massey V., Ghisla S. (1974). Role of charge-transfer interactions in flavoprotein catalysis. Ann. N. Y. Acad. Sci.

[42] Ghisla S. (1980). Fluorescence and optical characteristics of reduced flavins and flavoproteins. Methods Enzymol.

[43] Song S.-H., Dick B., Penzkofer A. (2007). Photo-induced reduction of flavin mononucleotide in aqueous solutions. Chem. Phys.

[44] Penzkofer A., Stierl M., Hegemann P., Kateriya S. (2012). Absorption and fluorescence characteristics of photo-activated adenylate cyclase nano-clusters from the amoeboflagellate *Naegleria gruberi* NEG-M strain. Chem. Phys.

[45] Wahl P., Auchet J.C., Visser A.J.W.G., Müller F. (1974). Time resolved fluorescence of flavin adenine dinucleotide. FEBS Lett.

[46] Van den Berg P.A.W., Feenstra K.A., Mark A.E., Berendsen H.J.C., Visser A.J.W.G. (2002). Dynamic conformations of flavin adenine dinucleotide: Simulated molecular dynamics of the flavin cofactor related to the time-resolved fluorescence characteristics. J. Phys. Chem. B.

[47] Forssell P., Lahtinen R., Lahelin M., Myllärinen P. (2002). Oxygen permeability of amylose and amylopectin films. Carbonate Polym.

[48] Rankin J.C., Wolff I.A., Davis H.A., Rist C.E. (1958). Permeability of amylose film to moisture vapor, selected organic vapors, and the common gases. Ind. Eng. Chem.

[49] Turro N.J., Ramamurthy V., Scaiano J.C. (2009). Principles of Molecular Photochemistry. An Introduction.

[50] Penzkofer A., Simmel M., Riedl D. (2012). Room temperature phosphorescence lifetime and quantum yield of erythrosine B and rose bengal in aerobic alkaline aqueous solution. J. Lumin.

[51] Shirdel J., Penzkofer A., Procházka R., Shen Z., Daub J. (2007). Absorption and fluorescence spectroscopic characterisation of a phenothiazine-flavin dyad. Chem. Phys.

[52] Schweitzer C., Schmidt R. (2003). Physical mechanisms of generation and deactivation of singlet oxygen. Chem. Rev.

[53] Guillet J.E., Andrews M. (1992). Studies of oxygen diffusion in poly(styrene-*co*-1-naphthyl methacrylate) by phosphorescence quenching. Macromolecules.

[54] Gao Y., Baca A.M., Wang B., Ogilby P.R. (1994). Activation barriers for oxygen diffusion in polystyrene and polycarbonate glasses: Effects of low molecular weight additives. Macromolecules.

[55] Holzer W., Penzkofer A., Susdorf T., Álvarez M., Islam S.D.M., Hegemann P. (2004). Absorption and emission spectroscopic characterisation of the LOV2-domain of Phot from *Chlamydomonas reinhardtii* fused to a maltose binding protein. Chem. Phys.

[56] Masuda S., Bauer C.E. (2002). AppA is a blue light photoreceptor that antirepresses photosynthesis gene expression in *Rhodobacter sphaeroides*. Cell.

[57] Penzkofer A., Stierl M., Hegemann P., Kateriya S. (2011). Photo-dynamics of the BLUF domain containing soluble adenylate cyclase (nPAC) from the amoeboflagellate *Naegleria gruberi* NEG-M strain. Chem. Phys.

[58] Iseki M., Matsunaga S., Murakami A., Ohno K., Shiga K., Yoshida K., Sugai M., Takahashi T., Hori T., Watanabe M. (2002). A blue-light-activated adenylyl cyclase mediates photoavoidance in *Euglena gracilis*. Nature.

[59] Zirak P., Penzkofer A., Lehmpfuhl C., Mathes T., Hegemann P. (2007). Absorption and emission spectroscopic characterization of blue-light receptor Slr1694 from *Synechocystis* sp. PCC6803. J. Photochem. Photobiol. B.

[60] Shirdel J., Zirak P., Penzkofer A., Breitkreuz H., Wolf E. (2008). Absorption and fluorescence spectroscopic characterisation of the circadian blue-light photoreceptor cryptochrome from *Drosophila melanogaster* (dCry). Chem. Phys.

[61] Song S.-H., Dick B., Penzkofer A., Pokorny R., Batschauer A., Essen L.-O. (2006). Absorption and fluorescence spectroscopic characterization of cryptochrome 3 from *Arabidopsis thaliana*. J. Photochem. Photobiol. B.

[62] Van de Linde S., Krstić I., Prisner T., Doose S., Heilmann M., Sauer M. (2011). Photoinduced formation of reversible dye radicals and their impact on super-resolution imaging. Photochem. Photobiol. Sci.

[63] Heilemann M., van de Linde S., Mukherjee A., Sauer M. (2008). Super-resolution imaging with small organic fluorophores. Angew. Chem. Int. Ed.

[64] Penzkofer A., Bansal A.K., Song S.-H., Dick B. (2007). Fluorescence quenching of flavins by reductive agents. Chem. Phys.

[65] Holmström B., Oster G. (1961). Riboflavin as an electron donor in photochemical reactions. J. Am. Chem. Soc.

[66] Moore W.M., Spence J.T., Raymond F.A., Colson S.D. (1963). Photochemistry of riboflavin. I. The hydrogen transfer process in the anaerobic photobleaching of flavins. J. Am. Chem. Soc.

[67] Holmström B. (1964). The mechanism of the photoreduction of riboflavin. Arkiv Kemi.

[68] Radda G.K., Calvin M. (1964). Chemical and photochemical reductions of flavin nucleotides and analogs. Biochemistry.

[69] Zulkowsky K. (1880). Verhalten der Stärke gegen Glycerin. Ber. Deutsch. Chem. Ges.

[70] Zirak P., Penzkofer A., Mathes T., Hegemann P. (2009). Photo-dynamics of roseoflavin and riboflavin in aqueous and organic solvents. Chem. Phys.

